# A Novel Point-of-Care BioNanoSensor for Rapid HIV Detection and Treatment Monitoring

**DOI:** 10.4172/2155-6113.1000454

**Published:** 2015-05-08

**Authors:** Tomasz Rozmyslowicz, Johann deSa, Ryszard Lec, Glen N Gaulton

**Affiliations:** 1 Department of Pathology and Laboratory Medicine, University of Pennsylvania, School of Medicine, Philadelphia, USA; 2 School of Biomedical Engineering, Science and Health Systems, Drexel University, Philadelphia, USA

**Keywords:** HIV-1 infection, HIV detection, BioNanoSensors, Piezoelectric high frequency technology, Electronic Measurement System, Polyclonal sheep anti-HIV-1 gp120, HIV treatment monitoring

## Abstract

We report here a new diagnostic approach to the direct detection of HIV in blood or other body fluids that is rapid, sensitive and potentially applicable in a point-of-care setting. The approach follows on the development of a novel BioNanoSensor (BNS) device that utilizes piezoelectric technology to detect the presence of the HIV surface glycoprotein gp120 in a nanoscale format. The detection range of the BNS device for the biomarker gp120 displayed a low-end sensitivity of 6.5×10^4^ HIV viral particles/ml, while using a small fluid sample (5 µl) and with a reaction time of less then 30 seconds. Performance of this device indicated that the BNS has utility for direct detection of HIV particles prior to, and independent from, antibody formation. Accordingly, this device holds utility to monitor the status of HIV infection both early after exposure to virus as well as during chronic HIV infection. The BNS parameters of small sample volume, compact device size, and detection sensitivity indicate that the BNS is potentially useful in the point-of-care and/or home setting for monitoring decisions regarding HIV treatment on a real-time basis.

## Introduction

The application of highly active antiretroviral therapy (HAART) has markedly improved care and prolonged life for many individuals infected with HIV. As a result, those living with chronic HIV infection and/or AIDS are now estimated at 35.3 million worldwide [1]. More specifically, in North America, and Western and Central Europe the number of HIV infected individuals nearly doubled in the last six years to an estimated 2.3 million [1,2]. The vast majority of these individuals require both continual access to HAART and regular monitoring of their HIV load to ensure control of infection status. Additionally, as the life expectancy of infected individuals increases, complicating effects of HIV on multiple tissues and organ systems are emerging, which both enhances the potential for treatment complications and diminishes positive medical outcomes [3,4]. Given that the number of persons infected with HIV is forecast to grow steadily in the years to come, treatment strategies will need to significantly improve in order to better accommodate the desired paradigms for improved care. The ability to monitor HIV infection through diagnostic approaches that are at once sensitive and reproducible as well as cost effective and user friendly is critical to this end.

The natural history of untreated HIV infection typically encompasses three periods: primary infection (acute and early), chronic infection, and progression to AIDS with advanced HIV infection. Early HIV infection is characterized by a period of rapid viral replication in which the level of viral RNA level typically exceeds 100,000 copies/ml. During chronic infection virus level decline. Often to a steady state of more than 1000 copies/ml). Left untreated, in the vast majority of individuals, HIV levels eventually rebound to levels seen during the initial phase of infection coincident with the clinical progression to AIDS. In contrast, treatment with anti-viral therapy (HAART/ART) is often effective in suppressing serum viral RNA levels below 1,000 copies/ml or indeed may be undetectable [5–9]. The importance of a device with sensitivity spanning this range of virus concentrations is critical for effective clinical management.

Available HIV screening methods are limited by cost, portability and/or detection platform. Assessment of HIV exposure by antibody screening can be determined using a commercial, over-the-counter kit; however, this method is not reliably sensitive until weeks after exposure when the immune response to HIV is reliably induced [10–12], and is not suitable for direct measurement of HIV virus or of monitoring HIV infection levels over time, as antibodies to HIV are measured not HIV particles. More sensitive, quantitative tests to detect virus directly, using either ELISA or PCR-based methods, require dedicated clinical laboratories and personnel and are accordingly more costly. The recently marketed *Alere Determine™ HIV-1/2Ag/Ab Combo* test and PCR-based *Alere™ q HIV-1/2 Detect* screening method offer important improvements on these approaches, nonetheless the need remains for an in-home test to measure the presence of HIV that is both quantitative and affordable [13]. Our intent is to develop a diagnostic assay for the direct measurement of HIV in body fluids that is quantitative, sensitive, portable and of low cost thereby facilitating in-home use. An additional advantage of our design is that the device platform is expandable for use in co-monitoring other medically related or co-morbid conditions to HIV infection [14–16].

Recent progress in micro-electronic and micro-mechanical fabrication technologies opens exciting avenues for the development of a new class of devices to measure chemical and biological elements [17]. High frequency BioNanoSensors (BNS) are small (microchip size), solid-state devices with disk, plate or prism shapes that are implanted with a system of metal electrodes used for interfacing the sensor with electronic circuits. They are label-free, inexpensive, portable and simple to use, and can sense gases, fluids and solid materials with high accuracy and reproducibility; thus, they are well suited for applications in analytical labs as well in point-of-care settings. Among several BNS detection systems, piezoelectric high frequency technology provides a particularly attractive platform. These devices are compatible with integrated circuit, and micro and nanoelectromechanical systems, show excellent aging characteristics, and are capable of measuring multiple components in one sensor package. Sensors based on this technology can be manufactured using standard photolithography, and hence, are relatively inexpensive to produce. Piezoelectric sensors function as resonant electromechanical units that can be excited at their fundamental and harmonic frequencies to generate acoustic waves having different penetration depths. This sensing attribute provides the distinctive capability of ‘slicing’ biological interfaces simultaneously at different depths, thus improving selectivity, sensitivity and reliability during detection.

Of the many types of piezoelectric sensors [18], the thickness shear mode (TSM) resonator [19], the acoustic plate mode (APM) device [20] and the surface skimming bulk wave (SSBW) device [21] generate pure shear motion, and have been used for fluid sensing, however, piezoelectric sensors have also been used to detect gases [22], viscoelastic properties of liquids [23,24], electrochemical processes in solutions [25], and to characterize superhydrophobic materials and interactions between solid particles [26]. More specifically, TSM sensors have been used to monitor biological processes such as cell-surface interactions [27] and adsorption/desorption [28]. A number of piezoelectric biosensors have been developed for medical applications, a common approach being immunosensors in which antibodies or antigens are immobilized on the TSM sensor surface [29]. Examples of this approach include the measurement of microorganisms, cells and toxins including herpes virus [30], detection of other viruses including hepatitis [31], African swine fever [32], Coxsackie B4 and, Hantavirus [33], and preliminarily HIV-1 and HIV-2 [34].

We describe herein the development of a novel BNS based on TSM-immunosensor technology [35] that is capable of directly measuring the HIV surface glycoprotein gp120 in human plasma. Housed in a portable and disposable device the detection reaction requires small fluid sample (5 µl), a reaction time less then 30 seconds, is specific to gp120, and is sensitive at the nanogram scale.

## Materials and Methods

### The sensor device hardware

A key element of the BNS is the TSM sensing microstructure. The important TSM working parameters include the operational frequency, the dynamic range and the noise level. The operational frequency is dependent on the membrane thickness of the sensor. The dynamic range and the noise level are determined by the Q-factor of the TSM, which in turn is affected by the roughness, the flatness, and the level of defects in the membrane. Sensors used here were fabricated using a dedicated integrated circuit microfabrication process [36,37]. Piezoelectric materials (quartz) was cut and polished to the required thickness and shape. The masks for the given electrode pattern were developed and the metal electrodes were either RF sputtered or made photolithographically. High frequency sensors, above 50 MHz, were made using a combination of reactive ion and chemical wet etching techniques. Electrical connections were made using ultrasonic bonders.

### Preparation and use of the sensor surface

The gold electrode surface of TSM sensors was cleaned using Piranha solution (one part of 30% H_2_O_2_ in three parts H_2_SO_4_) [38]. After 2 min exposure time, the sensor surface was rinsed with distilled water and dried in a stream of nitrogen gas. To adhere antibody, or other protein, the cleaned TSM sensor was first rinsed with phosphate buffer saline (PBS) and, once the frequency and magnitude responses were stabile, an aliquot (5 µl) of the capture antibody (anti-gp120 or control) was introduced to the sensor surface at a concentration of 1mg/ml which provides full coverage of the sensor surface.

Polyclonal sheep anti-HIV-1 gp120 was obtained from Aalto Bio Reagents (Dublin, Ireland). Anti-gp120 was prepared following immunization with a conserved domain of 15 amino acids within the HIV-1 envelope, and thus, recognizes conserved gp120 epitopes across all HIV subtypes isolated to date [39]. This is an important feature, as it is well known that the HIV-1 envelope protein gp120 displays considerable sequence variation in nature. The frequency and magnitude change due to the adsorption of the antibody, blocking reagent and, finally, gp120 or HIV on the surface of electrode was monitored as a function of time. Reagent concentration and tracing duration are described in Results. In brief, BNS reactions were measured following the addition (5 µl-5 ml) of either purified gp120 (1pg-100ng, Aalto Bio Reagents), pseudotyped virus particles or whole serum. Replication defective HIV particles were pseudotyped with or without gp120 ENV: NL43/R3A (ENV+) and NL43 (ENV-), as described previously [40–42]. In brief, the virus backbone is HIV-1 NL43, which is defective in both the Env and Vpr genes. The NL43/R3A (ENV+) virus was constructed by co-transfection gp120 Env isolated from R3A HIV. Serum from HIV positive and negative individuals was obtained from the University of Pennsylvania Center for AIDS Research core facility, and were characterized for virus concentration by RT-PCR using the bioMerieux NucliSens EasyQ HIV- 1v1.1 assay (bioMerieux, Inc, Durham, NC). The concentration of virus in these samples ranged from 0.14 – 1.6×10^6^ copies/ml). Prior to use, virus particles were rendered noninfectious by heat treatment for 2hr at 60°C with loss of infectivity confirmed by RT analysis.

### Signal detection

Alternating voltage was first applied to the TSM sensor to produce standing acoustic waves and enabling the sensor to function as a highly sensitive electromechanical resonator, transmitting a shear wave into the liquid medium. The shear wave penetrates liquid over a very short distance, on the order of tens to hundreds of nanometers, and the influence of the boundary (interfacial) conditions on the behavior of the sensor is very strong. The shear acoustic wave decays rapidly with the rate determined by the penetration depth factor, which is proportional to liquid viscosity and inversely proportional to liquid density and the frequency of the wave. By changing the frequency, one can inversely control the distance at which the wave probes the sensor-liquid interface. For example, at 5 MHz in PBS, the depth of penetration is about 280 nanometers, while at 500 MHz penetration is 26 nanometers. Usually, bionanosensors operate in the range from single MHz to several hundreds MHz, and with multiresonant operation across this range the BNS can in effect ’slice’ the depth of penetration thereby improving the sensor performance in term of sensitivity, selectivity and resolution. Frequencies of 100–200MHz were used in our analysis.

We measured the electrical response of the TSM sensor, calculated by the electronic detection system in the vicinity of operating frequency range and the change in the frequency as a function of time. The magnitude of the response, the S21 scattering parameter, is defined as |S21|=20 log (100/(100+Zt)), and Zt=total electromechanical impedance of the TSM sensor that is a function of the liquid loading. When the BNS is loaded with a biological media the sensor response S21 will exhibit a shift in resonant frequency and a decrease in magnitude. Depending on the antibody-antigen interactions at the interface of the sensor surface and medium, a positive or negative shift can be seen in the frequency response [36]. These changes can be correlated to the mass accumulation on the sensor interface due to the binding of antigen (gp120) to the sensor probe antibody.

### The electronic measurement system

The measurement system used was based on the network analyzer technique and a personal computer for data acquisition and signal processing. The main feature of this technique is measurement of the trans-impedance of the TSM sensors. The network analyzer-based method allows for rapid and wide frequency band scanning of the trans-impedance characteristics of the TSM sensor. The time and frequency domain signatures of the TSM response to antibody-antigen interactions and the time characteristics (kinetics) were also obtained. The TSM sensors were measured as a one-port or two-port device depending on the specific biological measurement requirements. All sensors with their enclosures (the chamber, reference liquid, cables, etc.) were precalibrated in order to eliminate the influence of ambient conditions on the results. The measured sensor parameters were used for data processing and subsequent biological interpretation included the sensor resonant frequency, magnitude, phase, impedance, and their signatures in the time domain. For the sensor array, a system of electronically controlled microwave switches was used to change between different TSM sensors.

### Statistical analysis

Arithmetic means and standard deviations were performed using Microsoft Excel 2008. Statistical analysis of our data was analyzed using the Student t-test for paired and unpaired samples. Statistical significance was defined as p<0.05.

The acquisition and use of human samples was reviewed and approved by the University of Pennsylvania Institutional Review Board (IRB) – protocols: #803567 and # 809496.

## Results

### Detection of HIV-1 gp120

**Figure 1:**
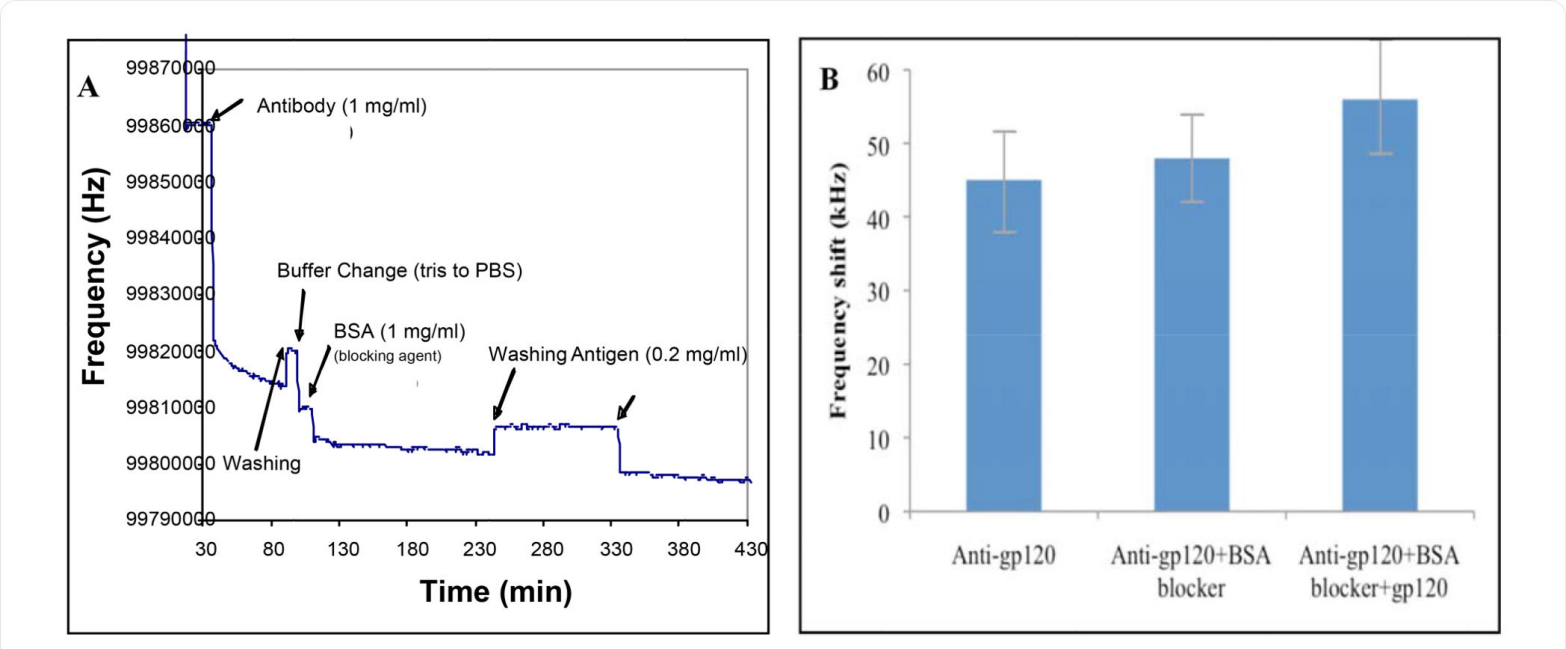
Response of the BNS sensor to gp120 binding: (**A**) The sensor resonant frequency change following sequential steps of the immobilization process and sensor exposure to gp120. **(B)** The difference in frequency response after each successive step of the BNS reaction.

An assessment of the capacity and sensitivity for the BNS device to detect HIV was first conducted by measuring the sensor wave deflection in response to binding of the major HIV surface glycoprotein gp120. In brief, the reaction was conducted using a TSM surface first coated with polyclonal sheep anti-HIV-1 and then probed with a commercial preparation of gp120. To initiate the experiment, a 5 mm diameter, 16 mm thick 100/200 MHz quartz crystal with deposited 1.5/0.7 mm diameter gold electrodes was placed in a custom fabricated sensor holder. As shown in Figure 1A, following the sensor assembly binding inflection was monitored continuously at a frequency of 100/200 MHz as a series of 5 µl blocking and sample solutions were placed in contact with the sensor. Reference measurements were first taken using TRIS buffer (starting point measurement). Antibody solution (anti-gp120 at 1 mg/ml, dissolved in PBS) was then added and incubated for 60 minutes to allow for antibody binding to the sensor surface using a standard chemi-adsorption procedure: note initial negative resonance deflection. The antibody-coated sensor was then gently rinsed with TRIS buffer (in concentration of 20 mmol/l and pH 7.6), followed by a 15-minute incubation in PBS to gain reference measurements. Bovine serum albumin (BSA) (1 mg/ml, dissolved in PBS) was then adsorbed to the sensor surface for 1 hour to block any remaining binding sites. Residual BSA was washed off in PBS, and the sensor was then probed by the addition of 5 µl of gp120 at 0.2 mg/ml (1mg total protein, dissolved in PBS), and monitored over the next 60 min. An increase in BNS frequency response of 5–8,000 Hz was consistently observed following the sequential addition of antibody, antibody/blocker, and then antibody/blocker in the presence of gp120 as shown in Figure 1B.

**Figure 2:**
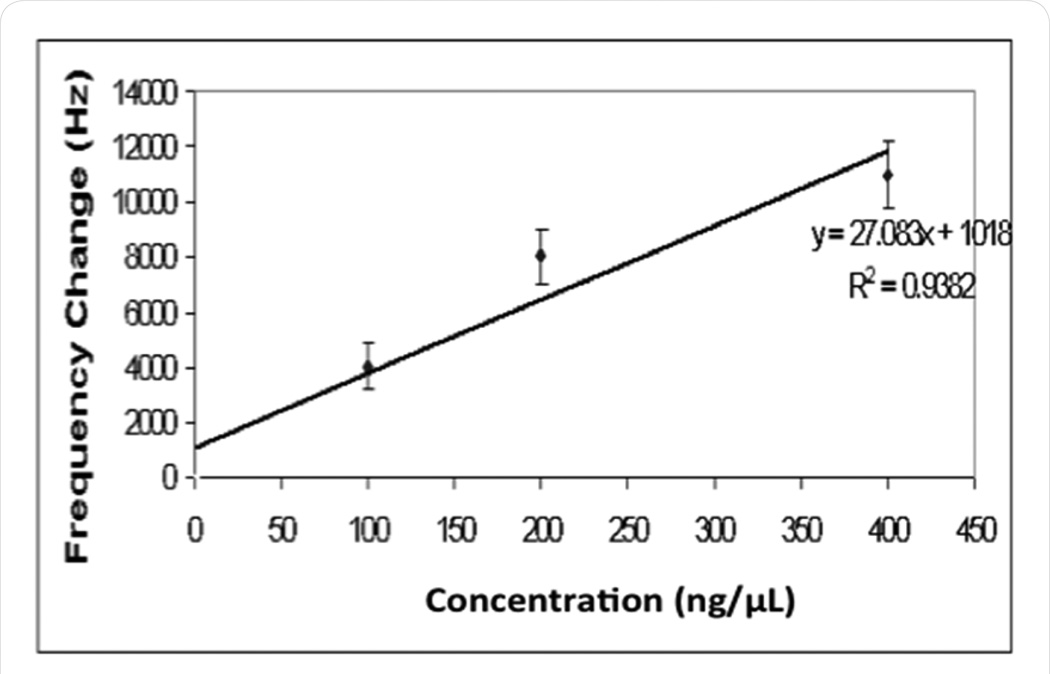
Frequency response change to gp120 binding: The magnitude of sensor frequency change was measured following the addition of gp120, over the range of 0.1–0.4 mg/ml, to the BNS device.

The sensitivity of the BNS for gp120 detection was next measured by mapping responses to a serial dilution of gp120 concentrations (0.1 – 0.4 µg/µl). The resulting sensitivity curve, shown in Figure 2, predicts a current lower limit of BNS detection at 25 ng/µl gp120.

### Detection of intact HIV virions

**Figure 3:**
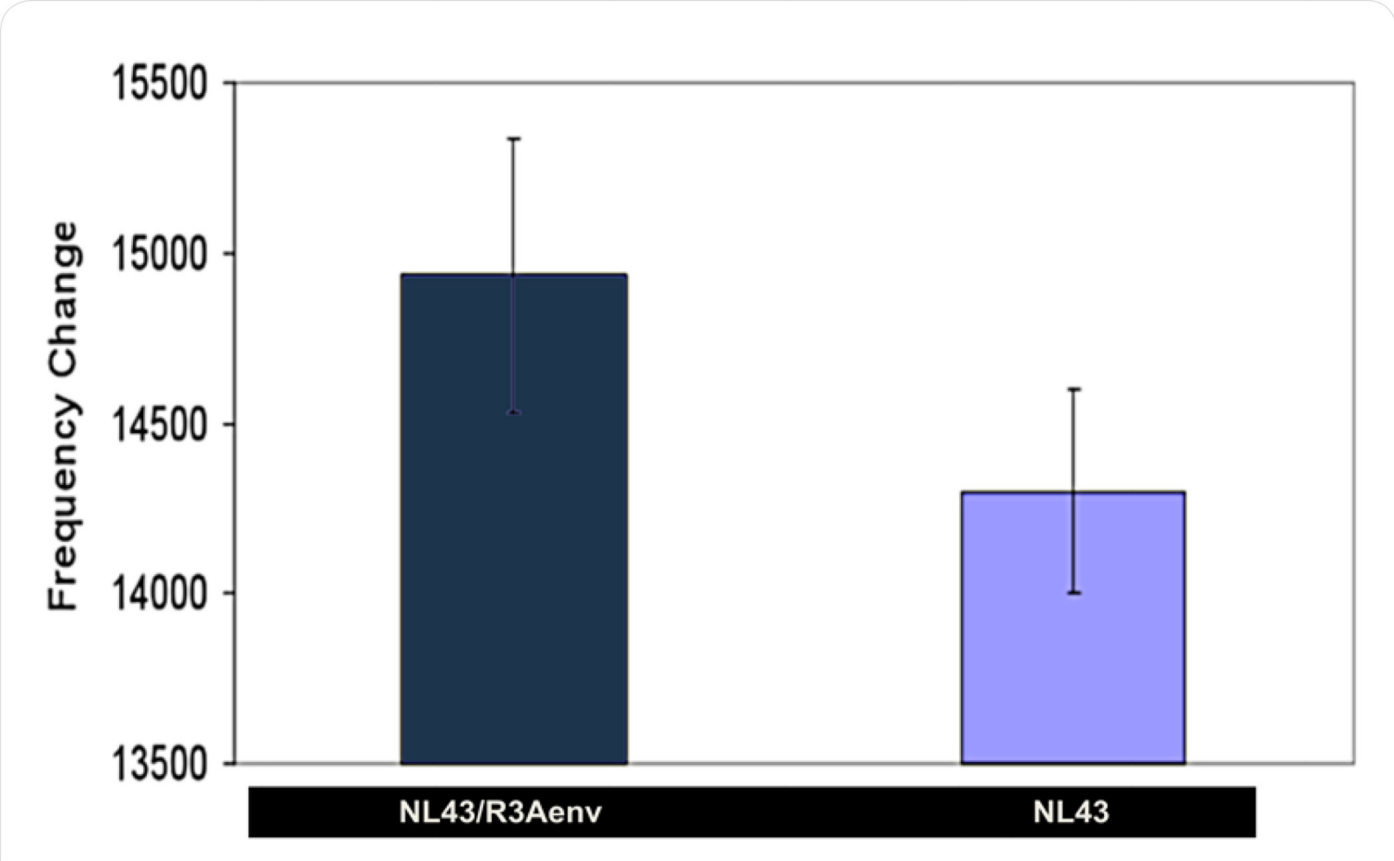
BNS resonant frequency change in response to HIV: gp120 positive NL43/R3Aenv virus and gp120 negative NL43 virus.

As a prime goal for a portable BNS sensor is the direct measurement of virus in blood or body fluids, we next analyzed the capacity of the BNS device to detect intact HIV-1 virions using gp120 as the binding vehicle. To simultaneously control for the specificity of gp120 binding on whole virus we contrasted BNS responses to the presence of two forms of HIV-1 - pseudotyped with or without gp120. The backbone of these viruses is HIV-1 NL43, which is modified to be genetically defective for both Env and Vpr gene function. The negative control for the experiment was thus, naïve NL43 virus lacking Env gp120; whereas, the test sample was NL43 virus pseudotyped with gp120 Env isolated from the HIV strain R3A [40]. Assays to measure the BNS response to NL43 virus and the NL43/R3Aenv pseudotyped virus (each at 78ng/ml) were conducted as described above, using again sensors coated with polyclonal anti-gp120. Results for the detection of gp120 are shown in Figure 3. All experiments were run in triplicate, the average and standard deviation of which is plotted. A significant shift in the resonance frequency was consistently seen with the gp120 positive pseudotyped NL43/R3Aenv virus as contrasted to gp120 negative NL43.

### BNS detection of HIV virus in human plasma

**Figure 4:**
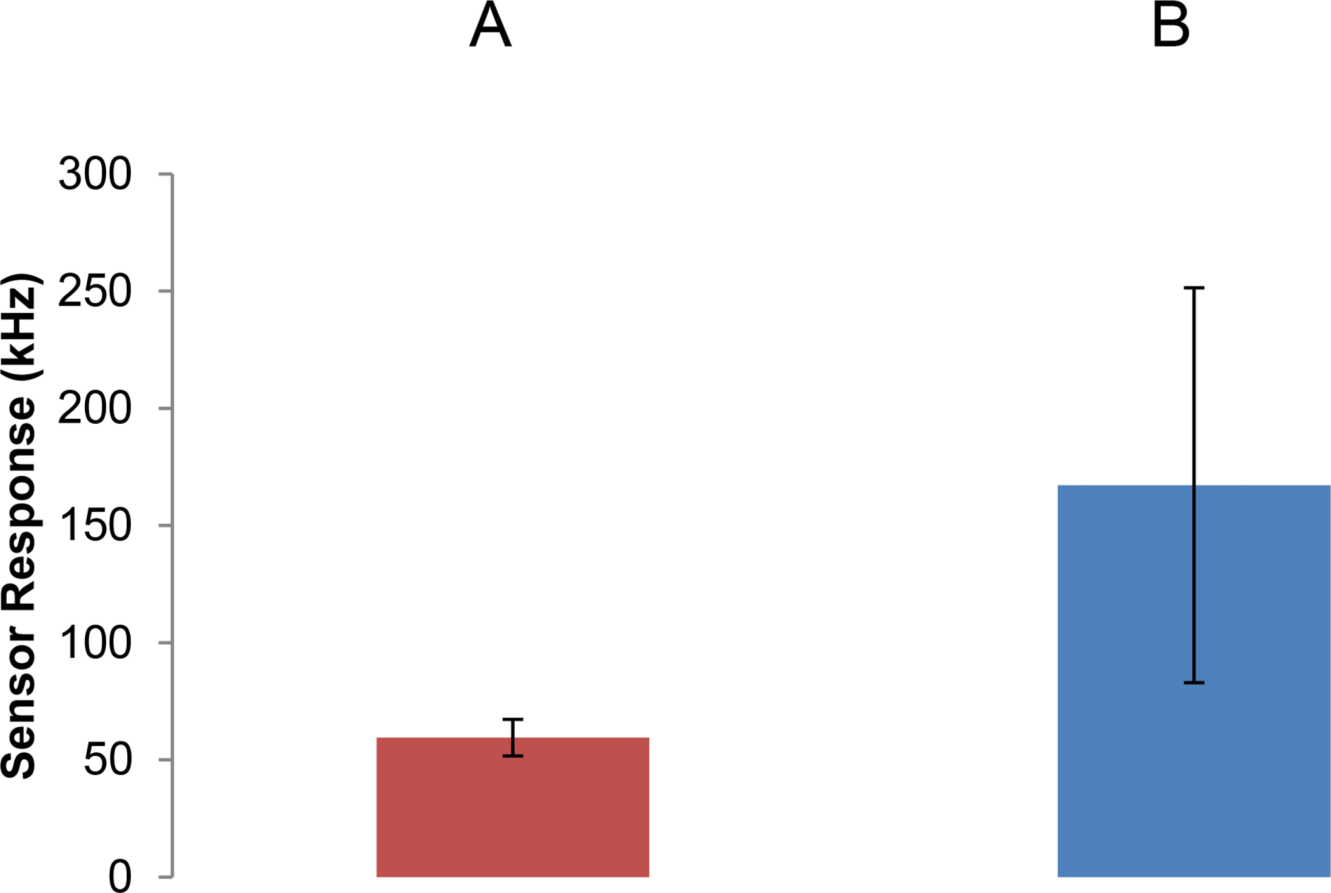
BNS resonant frequency change in response to HIV-1 in human plasma: Plasma samples were obtained from 10 seropositive individuals and the BNS response measured using a sensor loaded with either (**A**) blocked by addition of BSA/no antibody or (**B**) anti-gp120. Histograms represent the sensor response inflection at 200MHz.

With knowledge of both BNS binding specificity and sensitivity to gp120 in hand, we extended our analysis to the direct detection of HIV-1 in plasma samples obtained from individuals infected with HIV-1. So that these experiments might be conducted without unnecessary health risks, samples were first rendered replication defective by heat treatment. The BNS frequency change in samples from HIV-1 seropositive individuals, as contrasted to control, is shown in Figure 4. For this analysis, plasma aliquots obtained from ten seropositive individuals were analyzed in parallel using the BNS device in either the presence or absence of detecting anti-gp120 antibody. The virus load of these samples ranged from 0.14–1.6×10^6^ copies/ml.

**Figure 5:**
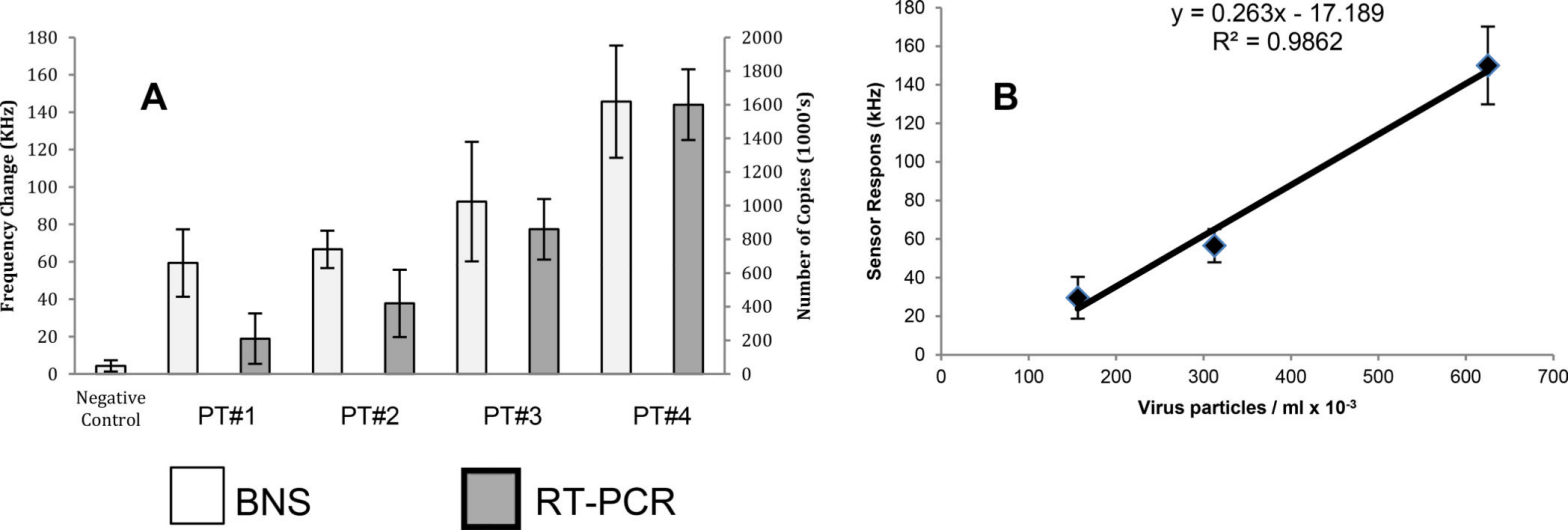
Comparative sensitivity analysis of HIV detection in plasma: (**A**) BNS response to HIV positive and negative human plasma samples (PT). BNS frequency shift in kHz using 200MHz sensors (left reference ordinate) and virus copy number by RT-PCR (right reference ordinate). (**B**) Linear regression analysis of sensor response with virus particle concentration. The data exhibits a correlation coefficient of 0.9862.

To more fully characterize the sensitivity of the BNS device for plasma HIV detection we simultaneously probed seropositive plasma aliquots to assess the readout of assays performed using either the BNS device or the RT-PCR reaction. Shown in Figure 5A is the virus load for each sample, as determined by RT-PCR assay, as well as the BSN response to that sample. Results shown in Figure 5B plot the sensor response relative to virus concentration. The results demonstrate a clear correlation between BNS signal intensity and HIV-1 load within the range of 0.14–1.6×10^6^ particles/ml. Linear regression analysis of these results, shown in Figure 5B, predicts a lower detection limit of 6.5×10^4^ virus particles/ml.

## Discussion

The results of our studies demonstrate the feasibility of BNS technology for specific detection of HIV particles. In brief, we observed: (1) measurement of nanogram quantities of gp120 HIV protein; (2) specificity for gp120 detection in both free protein and intact virus particles; and (3) measurement of whole virus particles in seropositive human plasma with a linear correlation to RT-PCR determination of virus load, and at a predicted lower BNS detection sensitivity of 6.5×10^4^ particles/ml. Importantly, BNS assays were conducted with small fluid sample (5 µl-5 ml) in a 10–20 mm^3^-detection chamber, and results were obtained within 30 seconds of initiation. These results support the potential of BNS technology to address the vexing problem of manufacturing a small, portable and inexpensive device capable of directly detecting HIV in blood and other body fluids.

There is an urgent need to develop a portable and inexpensive diagnostic test for early, direct detection of HIV. The applicability of such a device includes HIV diagnosis following potential exposure, the tracking of virus levels in infected individuals and, in a related manner, the management of AIDS-related disease progression in patients undergoing HIV therapies such as HAART. We have made significant progress toward this goal by validating detection of the HIV biomarker gp120 using the BNS device. While there are many advantages to this approach, the primary current limitation to HIV detection using BNS is detection sensitivity: to have widespread clinical utility and commercial viability, the BNS must reliably detect HIV at ~1×10^3^ particles/ml, a 1.5 order of magnitude improvement over current measurements. Approaches to increasing the detection sensitivity include advancement of the sensor micro-fabrication technology and sensor electrode geometry, modification of the measurement technique used to detect small changes in the operational frequency of the sensor, and altering properties of the biological sensing surface interface to increase binding probe density and/or signal amplification.

Since the BNS system is label-free and detects the physical presence of chemical species adsorbed on the transducer surface, nonspecific binding might also interfere more in this format than for other devices. This is being addressed by exploring more robust blocking techniques and agents. Similarly, the BNS fabrication processes may introduce limitations as quartz etching, photo/hard etch masking, and metal step coverage may introduce deep wells, as well as long and deep microfluidic line structures. If necessary, a combination of wet/dry etching techniques can be used as an alternative method for fabrication and we believe that this will provide good step coverage, fewer defects, as well as a flat, smooth surface [37]. While these are potential limitations to the widespread application of a BNS device, the inherent technology of BNS provides an advantage over other diagnostic technologies through the capacity to monitor the kinetics of target detection. In this instance one would measure the full time course of gp120 or virus binding to the detection surface. Such data provides a valuable asset for a commercial test in that a comparison of kinetics between a reference standard of reaction kinetics and/or sequential reactions for one patient provides an inherent quality control of the assay processes.

The results described in this paper hold promise for development of a new form of diagnostic assay – the multicomponent BNS. The promise of this approach is extraordinary, both to provide a reliable and portable HIV diagnostic, as well as to enable sensing of multiple co-morbid disease biomarkers. The most immediate application of this approach lies with meeting the needs of less mobile populations and in settings that do not have ready access to well equipped health clinics. In a final configuration, a hand-held BNS device would launch a sharp needle marginally through the epidermis of the finger in order to allow a small amount of blood (~10–100 µl) to enter onto a disposable testing strip that is coupled to a biochip housing the BNS sensor, piezoelectric transducer and fluidic nanosystem. The sensor would be coupled to an electronic interface complete with a digital LCD monitor. Much like a portable blood glucose measuring apparatus, the BNS device would then enable periodic assessment of HIV status administered by the infected person her/himself and shared through remote monitoring to a healthcare professional. Lastly, these results also point to the broader utility of the BNS platform and sensing design in detecting other metabolites, separately or in parallel to HIV, for which antibody or other binding reagents exist. Examples include the measurement of other less mutable core HIV components such as the p24 protein, cellular markers such as CD4, and blood metabolites such as hemoglobin all of which are important to monitor during active infection.
